# The Effects of Adipocytes on the Regulation of Breast Cancer in the Tumor Microenvironment: An Update

**DOI:** 10.3390/cells8080857

**Published:** 2019-08-08

**Authors:** Dinh-Toi Chu, Thuy Nguyen Thi Phuong, Nguyen Le Bao Tien, Dang-Khoa Tran, Tran-Thuy Nguyen, Vo Van Thanh, Thuy Luu Quang, Le Bui Minh, Van Huy Pham, Vo Truong Nhu Ngoc, Kushi Kushekhar, Thien Chu-Dinh

**Affiliations:** 1 Faculty of Biology, Hanoi National University of Education, Hanoi 100000, Vietnam; 2 School of Odonto Stomatology, Hanoi Medical University, Hanoi 100000, Vietnam; 3 Former address: Centre for Molecular Medicine Norway (NCMM), Nordic EMBL Partnership, University of Oslo and Oslo University Hospital, 0349 Oslo, Norway; 4 Department of Animal Science, College of Agriculture and Life Science, Chonnam National University, Gwangju 61186, Korea; 5 Institute of Orthopaedics and Trauma Surgery, Viet Duc Hospital, Hanoi 100000, Vietnam; 6 Department of Anatomy, University of Medicine Pham Ngoc Thach, Ho Chi Minh City 700000, Vietnam; 7 Department of Cardiovascular and Thoracic Surgery, Cardiovascular Center, E Hospital, Hanoi 100000, Vietnam; 8 School of Medicine and Pharmacy, Vietnam National University, Hanoi 100000, Vietnam; 9 Department of Surgery, Hanoi Medical University, Hanoi 100000, Vietnam; 10 Center for Anesthesia and Surgical Intensive Care, Viet Duc Hospital, Hanoi 100000, Vietnam; 11 NTT Hi-tech Institute, Nguyen Tat Thanh University, 300A Nguyen Tat Thanh St., Ward 13, District 4, Ho Chi Minh City 700000, Vietnam; 12 AI Lab, Faculty of Information Technology, Ton Duc Thang University, Ho Chi Minh City 700000, Vietnam; 13 Institute of Cancer Research, Oslo University Hospital, 0310 Oslo, Norway; 14 Institute for Research and Development, Duy Tan University, Danang 550000, Vietnam

**Keywords:** adipocytes, breast cancer, obesity, adipokines, hormones, tumor microenvironment

## Abstract

Obesity is a global pandemic and it is well evident that obesity is associated with the development of many disorders including many cancer types. Breast cancer is one of that associated with a high mortality rate. Adipocytes, a major cellular component in adipose tissue, are dysfunctional during obesity and also known to promote breast cancer development both in vitro and in vivo. Dysfunctional adipocytes can release metabolic substrates, adipokines, and cytokines, which promote proliferation, progression, invasion, and migration of breast cancer cells. The secretion of adipocytes can alter gene expression profile, induce inflammation and hypoxia, as well as inhibit apoptosis. It is known that excessive free fatty acids, cholesterol, triglycerides, hormones, leptin, interleukins, and chemokines upregulate breast cancer development. Interestingly, adiponectin is the only adipokine that has anti-tumor properties. Moreover, adipocytes are also related to chemotherapeutic resistance, resulting in the poorer outcome of treatment and advanced stages in breast cancer. Evaluation of the adipocyte secretion levels in the circulation can be useful for prognosis and evaluation of the effectiveness of cancer therapy in the patients. Therefore, understanding about functions of adipocytes as well as obesity in breast cancer may reveal novel targets that support the development of new anti-tumor therapy. In this systemic review, we summarize and update the effects of secreted factors by adipocytes on the regulation of breast cancer in the tumor microenvironment.

## 1. Introduction

It is reported that breast cancer is one of the leading cancer types with a high mortality rate, besides lung and skin cancer [1,2]. Among the 36 most common cancer types, breast cancer accounts for 11.6% of incident cases, followed by 6.6% of cancer death in 2018 [2]. In China, the proportional rate of breast cancer has been rapidly increasing and associated with socioeconomic status [3]. Incident rates in urban areas are twice higher than in rural areas, with rates at 0.034% and 0.017%, respectively [3]. In Brazil, about 0.06% of the female population was newly diagnosed with breast cancer in 2018. Japan lists the highest rate with about 0.043% (the data was recorded in 2009) [4]. According to U.S. breast cancer statistics, it was estimated that about 12% of females had a high potency on the development of breast cancer in their life. The number of incidental breast cancer cases will reach 268,600 and more than 41,000 people are predicted to die in 2019 [1]. There are approximately 3.1 million people having breast cancer who are currently being treated, as of January 2019 [2].

Several risk factors have been identified that contribute to the development of breast cancer, including both genetic as well as non-genetic factors. In most cancer cases, environmental factors combined with genetic factors play a major impact on the development of breast cancer [5]. Evidently, obesity has been related to the initiation, development, and mortality of many cancers, especially breast cancer [6,7,8,9,10,11]. A large cohort study reported that BMI was linked to 17 out of 22 most common cancers in UK adults, including uterus, thyroid, gallbladder, kidney, cervix, colon, ovarian, and post-menopausal cancers [12]. In women, obesity increases the risk of death from breast and reproductive organs cancer [13,14,15,16]. A recent finding suggested that high BMI can reversibly regulate breast cancer in pre- and post-menopausal women [17]. High BMI induces breast cancer in pre-menopausal women but inversely in post-menopausal women. Similarly, 34 other studies also confirmed the correlation between high BMI and the risk of breast cancer in post-menopausal women [18]. Additionally, a recent cohort study has shown that there is a 52% increase in the development of breast cancer in post-menopause women by obesity [19]. Moreover, the mortality rate caused by breast cancer is much higher in obese people compared to lean people [11]. Obesity is linked to a higher cost of hospital care after chemotherapy and breast surgery [20]. Additionally, several studies have demonstrated that high weight gain is more important than BMI value for breast cancer development, and weight loss may reduce the incident rate in post-menopause women [4]. However, 35% of breast cancer patients reported increasing their body weight from 1.4 to 5.0 kg during and after breast cancer treatment [11,20,21,22,23]. Overall, in addition to the risk of hypertension and cardiovascular diseases, obesity is of high concern in cancer, especially in breast cancer patients.

Adipocytes are the major cellular components of adipose tissue and play an important role in maintaining the energy balance. Any dysfunction in adipocyte function or the energy balance leads to overweight and obesity. There are three types of adipocytes classified based on the origin, structure, and function including white, brown, and brite adipocytes [24,25,26,27]. In general, white adipocytes are the main storage of energy, whereas brite/brown adipocytes with higher expression levels of uncoupling protein 1, a mitochondrial proton carrier (UCP1), can generate heat by thermogenesis process [24,25,26,27]. Dysfunctional adipose tissue can secrete excessive fatty acid, cholesterol, triglycerides, hormones, and adipokines that are linked to metabolic dysfunction, insulin resistance, and inferior outcome in cancer treatment [28,29,30,31]. There are more than 600 proteins released by adipose tissue, in which about 50 adipokines are produced mainly by adipocytes, which provides a novel pool of biomarkers for the study of metabolic diseases [32]. Accumulated fatty acids will be up-taken into the tumors and generate energy for tumor development through β-oxidation. Fatty acids are the major source of ATP in the tumor. Moreover, insulin resistance and high glucose level in serum can provide nutrients for cancer cell invasion [29,33]. Additionally, adipocytes can contribute to the resistance of the chemotherapeutic drug. In co-culture with fibroblast, adipocytes can switch-off anti-cancer drug effects by metabolizing the drug into a less effective secondary [34]. Adipocytes also release extracellular matrix proteins and recruit other neighbor cells such as macrophages and other immune cells, mimicking the immune infiltrates of the tumor [35].

Thus, fat mass and adipose-tissue mass are strongly correlated with obese status and breast cancer in post-menopausal women. Adipocytes can regulate the tumor microenvironment via secreting energy nutrients which contributes to the risk of breast cancer incidence, proliferation, and metastasis. Beside other obesity-related complications, physical and mental consequences are strongly associated with cancers incidence and proliferation [6,36]. Therefore, it is necessary to understand how adipose tissues and adipocytes can contribute to the risk of breast cancer and mortality.

## 2. Adipocytes Regulate Breast Cancer via Their Metabolic Substrates

Obesity is strongly related to dysfunctional metabolism in adipocytes leading to several chronic diseases. High levels of free fatty acids, cholesterol, glycerol, and triglycerides get accumulated in serum to impact on the breast tumor initiation, development, and migration process (Table 1) [9,37,38,39,40,41,42,43,44,45,46,47,48,49]. In vitro, co-culture of mature adipocytes with breast cancer cells increased the breast cancer proliferation, that strongly supports the notion that adipocytes directly impact cancer cells by their secreted factors [50].

Free fatty acids (FFA) are released from daily meals, which deposit as lipid droplets in the adipose tissue. Both saturated and unsaturated fatty acids are released from high-fat diet or in original obese status [51,52]. Inflammation-induced obesity is an essential mechanism in the development and invasion of breast cancer [28,38,39]. Saturated fatty acids can activate toll-like receptor 4 to amplify inflammation and contribute to angiogenesis and tumor progression [53]. Inflamed microenvironment promotes adipocyte cell death, recruits macrophages, and forms a crown-like structure (CLS) [28]. The number of CLS is nine times higher in cancer patients with obesity or overweight than lean breast cancer women (70% and 8.3%, respectively) [54]. In addition, the numbers of CLS are related to poor prognosis and the development of breast tumors [55]. A study demonstrates that induced inflammation in white adipocyte tissue and increased CLS reduced the survival rate in patients [55]. Furthermore, saturated fatty acids can activate NF-κB, leading TNF-α production that acts on the breast cancer cell proliferation, invasion, and metastasis [46,56]. Beside saturated fatty acids, unsaturated fatty acids also contribute to breast cancer progression and prognosis. Long-chain (n-6) fatty acids (linoleic acid) are well-known as pro-inflammatory factors, which induce the development of breast cancer [47], whereas other fatty acids reversely impact on the breast cancer patients [43,45,49]. FFAs induce breast cancer invasion by activating the epidermal growth factor receptor, GTP-binding protein, and protein kinase C pathway [57], and by controlling cell proliferation via phosphatidylinositol 3-kinase (PI3K) [58] and cell migration through free fatty acid receptor 1 and 4 and AKT pathway activation [59]. In vitro, David et al. stimulated breast cancer cells proliferation by supplement of FFAs on serum-free medium in a dose-dependent manner, linoleic acid can stimulate breast cancer cell growth at three times higher concentration than oleic acid (0.75 µg/mL) [47]. In contrast, Basil et al. also demonstrated that FFAs can induce apoptosis and lipid peroxidation in the human breast cancer cell culture [49]. Thus, supplement fish oil is proposed for pre-menopausal women to eliminate the risk of breast cancer.

In obesity, cholesterol is significantly induced and released into the bloodstream. However, whether the total cholesterol or high-density lipoprotein (HDL) and low-density lipoprotein (LDL) influence breast cancer growth remains elusive. A meta-analysis study has shown the inverse correlation between total cholesterol and HDL to the risk of breast cancer [60]. Both LDL and very-low-density lipoprotein (VLDL) exposure stimulates the development, migration, and invasion of breast cancer cell through activation PIK3/AKT pathway, especially VLDL promotes lung metastasis and angiogenic activity on the breast cancer cells [44]. Furthermore, hyperlipidemia positively associated with breast cancer risk and total survival rate regardless of BMI [61]. An analysis with over 664,000 women revealed that women above the age of 40 with high serum cholesterol have a 45% higher risk of development of new breast cancer and 40% lower the survival rate and treatment outcome of developed breast cancer [62]. Co-treatment with statins to lower cholesterol level showed the improvement in total survival rate and cancer-specific survival in cancer patients [63]. However, the protective effects of statin are still being considered. A recent meta-analysis study, which collected health data from over 823,000 post-menopausal volunteers, showed a similar invasive rate between former, current, and non-users of statin during treatment of breast cancer [64]. Oxysterol 27-hydroxycholesterol (27-OHC) is known as the metabolite substrate of cholesterol by Cytochrome P450 Family 27 Subfamily A Member 1 (CYP27A1) enzymes. High level of serum cholesterol corresponds to a high level of serum 27-OHC [42,65]. 27-OHC plays as a liver X factor [37] (LXR) and estrogen receptor (ER) agonist [66]. LXR activation lowers cholesterol accumulation and suppresses cell growth in both normal and breast cancer cells [53,67]. Whereas ER activation promotes breast cell proliferation in estrogen receptor-positive breast cancer (ER+) cells but not in negative cell types [41]. Considering the impacts of 27-OHC on ER+ breast cancer cells, Lu et al. conducted a case-control study and demonstrated that treatment 27-OHC on breast cancer differs by menopausal status. The opposite effects are seen in the pre-menopausal and post-menopausal status [68]. Thus, it is necessary to conduct additional studies on the effect of 27-OHC on breast cancer.

Additionally, adipose tissue also releases exosomes. Adipocyte exosome contains proteins related to fatty acid oxidation (FAO) [48]. Obesity induces exosomes modulating FAO and further contributes to tumor migration [48,69]. Other investigations also demonstrated that microRNAs that are released from adipose exosomes can support breast cancer tumor growth and invasive capacity [50,70]. Among 98 secreted miRNAs, miR-3184-5p is the most upregulated, whereas miR-181c-3p is the most downregulated one in breast cancer. Both target on FOXP4 and PPARα [50]. There are eight miRNAs associated with BMI value, where miR-191-5p, miR-17-5p were identified involving in tumor progression. In detail, miR-191-5p upregulated by 17β-estradiol protected ERα-positive tumors against apoptosis. miR-17-5p was inversely the impact of inflammatory cytokines, resulting in suppress tumor growth [71]. A recent paper has reported miR-144 and miR-126 secreted by adipocytes induced brown differentiation and tumor progression [72].

Matrix metalloproteinases (MMPs) family is known to play vital roles in the invasion and metastasis of tumor cells. MMP-9 is highly expressed in breast tumors compared to normal tissue. A high level of MMP-9 has been reported to have a positive correlation to brain metastases in breast cancer patients [73,74,75]. MMP-11 and MMP13 expression were also promising markers linked to the poor outcome of breast cancer which may be a novel target for the treatment of breast cancer [76].

## 3. Adipocytes Regulate Breast Cancer via Their Released Hormones

Obesity also affects breast cancer in women by secretion of some adipokines, which are also called as “released hormones”, especially estrogen, adiponectin, leptin, and insulin (Table 2) [77,78,79]. Estrogen and estrogen derivatives are significantly induced in post-menopausal obese women with increased BMI [80,81,82]. A random evaluation of post-menopausal women reported that there was a 35% higher estrogen level and 130% higher estradiol level in obese people compared to the low BMI/athletic ones [81]. Estrogen is mostly produced in the ovary in pre-menopausal women, but in adipose tissues in post-menopausal women [83]. Cytochrome P450 aromatase, catalyzed to biosynthesized estrogen by converting androgens to estrogens, is highly expressed in adipose tissue [84]. Therefore, estrogen levels in breast tumors associated with adipose tissue are 10 times higher than in the blood [85]. In addition, cytokines produced in adipose tissues can also promote cytochrome P450 aromatase secretion to induce estrogen biosynthesis [86,87,88]. Estrogen receptors (ERs) are important for estrogen function, ERα is linked to tumor cell proliferation whereas ERβ contributes to a favorable prognosis [89]. ERα positivity is related to obesity-induced breast cancer especially in post-menopausal women [90,91]. Estrogen-ERs complex, stabilized with a co-activator protein, interacts with estrogen response element site on DNA, leading to the regulation gene expression related to growth, differentiation, and other dysfunctions [92]. This complex can also bind to the promoter region to modify that gene expression [93]. Furthermore, estrogen can act by mediating the ER-membrane pathway including GPCR-like protein, G proteins, MAPK/ERK, and PI3K/AKT pathway [94,95,96]. These signaling pathways contribute to the proliferation and survival of breast cancer via increasing Bcl-2, cyclin D1, number of G0/G1 cells [97,98,99]. Lastly, estrogens promote breast cancer invasion and migration. In vitro, estrogen treatment remodels cytoskeleton and acts on GPCR-like protein and estrogen receptor signaling pathway to induce metastatic on breast cell [100,101].

Adiponectin is secreted from white adipocyte tissue before binding to specific receptors to act on insulin resistance, glucose uptake, and FFA oxidation. However, obese people have a lower expression of adiponectin receptors, that can cause adiponectin resistance [102]. In the report published in 2004, breast cancer women who have low serum adiponectin may have a higher risk of angiogenesis and metastasis [103]. In contrast, high adiponectin level can lower the incident rate of breast cancer in women [104]. However, a recent stratified case-control study revealed that adiponectin levels are not associated with breast cancer in pre-menopausal women but negatively affected post-menopausal women [105]. Adiponectin reduces insulin resistance and inflammation properties in obesity, which can improve breast tumor microenvironment [106,107]. In contrast to leptin, adiponectin decrease proliferation and stimulate the cell death program in breast cancer cells by several pathways including AMP-activated protein kinase, mTOR, and NF-κB pathways [108,109,110]. Morad et al. conducted an in vivo test in human breast tissue showing estrogen treatment increased leptin secretion, whereas tamoxifen treatment increased adiponectin and adiponectin/leptin ratio after six weeks treatment [111]. Adiponectin treatment reduces tumor size in ER-negative breast cancer but induces in ER-positive breast cancer because of the strong effects of estrogen on breast cells [112]. In summary, adiponectin can be considered for breast cancer therapy in combination with other drugs.

The adipokine, leptin is strongly accumulated in obesity and promotes the development of breast tumors [113,114]. Leptin acts directly on the leptin receptors, leading to inflammation in adipocyte microenvironment and increases the risk of metastatic potential [115]. Thus, the impact of leptin depends on site of leptin receptors expression. Distant leptin-derived metastatic in breast cancer is associated with 34% in the leptin receptor-positive patients, but none in the leptin receptor-negative patients [116]. Leptin activates ERα to stimulate estrogen-related breast cancer pathway, resulting in the proliferation and migration [117]. Furthermore, leptin can increase aromatase activity at high concentration and induce estradiol and estrogen in obese women [118]. Mechanically, activation of leptin receptor stimulates downstream ERK1/2, AP1, STAT3, PI3K, and MAPK pathways [117,119]. Additionally, leptin also promotes tumor growth/migration via VEGF signaling (endothelial growth factor signaling), HIF-1α stabilization, which induces hypoxia condition in tumors [119]. The authors also reported that leptin can promote pancreatic cancer burden by inducing MMP-13 production in human [119].

Insulin resistance is most common in obesity and results in hyperinsulinemia. High serum insulin level increases breast cancer growth and invasion [40,120,121] via activation of the PI3K pathway [122], and improved insulin resistance can reduce metastasis in mice [121]. High fasting insulin reduces survival rate, results in poor outcome to anticancer therapy [123], and induces a 2.4 times higher incident rate in post-menopausal women (the results were evaluated by random selection) [40]. In addition, insulin itself induces aromatase activity, which is important to estrogen synthesis in adipose tissue [124]. On the other hand, hyperinsulinemia is highly associated with the amount of insulin-like growth factor 1 (IGF-1) locally, thus, excess IGF-1 in tumor cells [122]. IGF-1 binds to selected receptors (IGF-1R) and upregulates MAPK signaling pathway, resulting in proliferation, development, and progression [125,126]. In the outcome of tamoxifen treatment, IGF-1R is considered to evaluate the effectiveness of therapy. Higher expression of IGF-1R implies poorer outcome and survival rate in breast cancer patients [127].

In addition, other hormones such as visfatin, plasminogen activator inhibitor-1(PAI-1), and resistin slightly contribute to breast cancer initiation and progression. High levels of serum resistin and visfatin were found in post-menopausal women with breast cancer, which correlated with tumor size and a high risk of lymph node metastasis [128]. Resistin promotes breast cancer development via enhanced toll-like receptor 4-mediated transition and NF-κB activation [129]. Secreted PAI-1 by adipose tissue contributes to increase proliferation, angiogenesis, cell migration, and decrease apoptosis, which supports tumor invasion in the breast [130].

Overall, the adipokines significantly contribute to tumor cell proliferation, progression, and migration, which influence the outcome of prognosis in breast cancer patients. Leptin/adiponectin ratio is highly induced in breast cancer, and more interestingly, the ratio correlated to the level of obesity. People with high BMI together with high leptin/adiponectin ratio have a significantly higher risk of breast cancer incidence and metastasis [131].

## 4. Adipocytes Regulate Breast Cancer via Released Cytokines

Cytokines, generally produced by immune cells, play a vital role in pro-inflammation, anti-infection, and other cellular signaling. Along with white adipocyte, there are immunocytes, vascular, and stromal cells which exist in the adipose tissue, which are involved in the cytokines production (Table 3) [136]. In addition, adipocytes can also secrete several cytokines to regulate the surrounding cells and itself. In vitro co-culture of adipocytes and breast cancer cells results in the secretion of cytokines into the culture supernatant. The findings showed five (IL6, IL8, IFNᵧ-inducible protein 10, CCL2, and CCL5) out of 200 cytokines which were significantly increased after one-week culture, with immature adipocytes having higher cytokine secretion potential than mature adipocytes. Cytokines, produced by immature adipocytes, were reported to increase tumor initiation and metastasis burden in breast cancer [137]. A follow-up study with 534 patients demonstrated IL6, IL8, and IL10 may consider as indicators for poor prognosis and metastatic of breast cancer [138]. Several investigations also emphasized IL6, IL8, CCL2, and CCL5 as stimulators of the survival, proliferation, and invasion of breast cancer cells [139,140,141,142]. Moreover, serum IL6, IL8, and TNFα are related to an advanced stage and metastatic status of breast cancers, whereas IL6 and IL8 are most favorable for prognosis [143].

High levels of TNFα causes chronic inflammation and insulin resistance, that in turn provides positive effects on the development of cancers [144,145]. TNFα functions by interacting with TNF receptors, including TNFRI and TNFRII [146]. Several studies were conducted to determine the effects of TNFα both in vitro and in vivo experiments and in clinical trials. In vitro, TNFα promotes cell development in both ER-positive and ER-negative breast cancer cell lines [147,148]. In ER-positive cell lines, TNFα inhibits apoptosis, but not in ER-negative cell lines [148]. The authors showed that TNFα can activate NF-κB, ERK, AKT, and JNK pathways which are necessary for cell proliferation [145,147,149,150] and positively modulate estrogen metabolic pathway, leading to high amounts of estrogen in tumors [151]. Furthermore, TNFα also promotes tumor migration by inducing cytokines such as MMP-9 [149] and chemokines receptors CCR9 and CCR5 [152]. In vivo, mice treated with TNFα developed tumors compared to control mice group with PBS treatment [147,150]. Blocking TNFα significantly reduces breast tumor size [153]. In addition, TNFα regulates interleukin synthesis, adiponectin secretion, and aromatase expression in adipose tissue [154,155,156].

Numerous reviews have also reported the strong association between interleukins and breast cancers, notably IL1β, IL6, and IL8 [142]. Firstly, IL1β activates inflammatory NF-κB signaling pathway in the tumor cells to promote the development of breast cancer [157,158]. More importantly, IL1β strongly linked to migration in breast cancer. IL1β increases the phosphorylation of focal adhesion kinases and expression of MMP9 which are related to the adhesion and migration of cancer cells [159]. IL1β also induces cyclooxygenase-2, hypoxia-inducible factor 1α which promotes angiogenesis, inflammation, and metastasis in cancer [158,160]. Secondly, together with TNFα, IL6 is a major inflammation inducer which activates STAT3, resulting in cancer progression [161]. Interestingly, abrogation of STAT3 signaling by knocking out the STAT3 gene did not influence the tumor formation but greatly suppressed the lung metastasis in mice [162]. A recent study reports that IL6 triggers the expression of VEGF promoted cancer cell multiplication [142]. Thus, it is strongly evident that STAT3 regulates metastasis. High circulating IL6 correlated with poorer prognosis and high risk of metastatic burden [163]. In vitro, over-expression IL6 induces genes involved in epithelial-mesenchymal transition and lowers E-cadherin, supporting that IL6 can regulate adhesion and migration of breast cancer cells [164]. Together with other cytokines, IL8 is angiogenic in cancers [139]. IL6 and IL8 promote oncogene Ras transformation, and then actively induce cell development by many signal pathway [137]. IL6, IL8, IL1β, IP10, CCL2, and CCL5 contribute to the activation of Src kinases and signal to NF-κB pathway [137,165]. The patients with better treatment outcome had significantly low levels of IL8 compared to that of pre-treated patients [138].

Chemokines are small protein molecules that regulate leucocyte transportation in response to inflammation and/or homeostatic conditions. Chemokines such as CCL2, CCL5, CCL4, and CXCL8 are also involved in the incidence and development of breast cancer [166,167]. CCL2 and CCL5 induce tumor-associated macrophages (TAMs), thereby inhibiting T-cell activation and promoting angiogenesis in breast tumors [140]. In addition, CXCL18 is highly accumulated in TAMs in breast tumors [168]. CXCL18 induces IL4, IL13, and IL10 in TAMs promoting initiation and invasion of breast carcinoma cells [169,170] via NF-κB and AP-1 pathways [166]. Both CCL2 and CCL4 suppress infiltration of macrophages and immune cells to the tumor site and are related to an advanced stage and poor prognosis [171,172,173]. CXCl10, also called IP10, stimulates the release of other chemokines, tumor progression, and metastasis via CRXR3 and activation of NF-κB signaling pathway [174,175]. Chemokines directly bind to chemokine receptors, alters the expression and/or function of secondary messengers to stimulate angiogenesis, suppress anti-tumor immune cells’ infiltration [166]. Chemokine receptors CXCR1, CXCR2, CXCR4, CXCR5, and CX3CR1 are critical in the recruitment of macrophage in the breast tumor microenvironment. Therefore, anti-chemokine receptors can bring the therapeutic potential for the treatment of breast cancer. In fact, CXCR1 and CXCR2 antagonists are widely researched on the treatment of autoimmune diseases and prevention metastasis cancer. The clinical trials for breast cancer therapy still need further investigations in individual therapy or combination with chemotherapy or immunotherapy [176]. Moreover, tumor microenvironment in breast cancer continuously produces chemokines that may develop to a higher stage of disease and metastasis [166].

Furthermore, adipocytes also impact on the surrounding cells in the breast tumor microenvironment including immune cells, cancer-associated fibroblasts, endothelial cells, and mesenchymal stem cells [177]. Adipocytes can act as immune regulatory cells. Adipocytes release minimal amounts of cytokines that enhance cytokines/chemokines production in immune cells at different levels in the tumor [178,179]. Damage-associated chemicals released by dead and dying adipocytes stimulate recruitment of macrophages and other immune cells, which can be observed by the presence of crown-like structure within adipocytes [31]. In addition, the secreted cytokines can stimulate differentiation of breast cancer mesenchymal stem cells into adipocytes as well as cancer-associated fibroblasts that amplifies the impacts of adipocytes in the tumor microenvironment. Cancer-associated fibroblasts, an important IL-6 source, strongly related to tumor growth and therapy resistance which can be targeted for the development of potential drugs on treatment and prevention of breast cancer [180,181]. CXCL12 secreted by cancer-associated fibroblasts promotes the proliferation of breast tumor cells. The level of serum CXCL12 is associated with a high mortality rate in cancer patients [182,183,184]. Adipocytes surrounding tumor cells exhibit phenotypical changes termed as adipocyte-derived fibroblasts (ADFs). ADFs enhance release fibronectin and collagen I, increase gene expression of adipokines and adipocytokines (TNF-α, IL-6 and IL-1β) which induce invasive abilities of breast tumor cells [185,186]. Among tumor microenvironment, endothelial cells are converted to fibroblast-like cells in the presence of TNF-α. These cells enhance the production of chemokines CXCL1/2 that promote tumor cells survival and metastasis as well as contribute to chemotherapeutic resistance [187]. It was reported that exosomes secreted by preadipocytes regulated early-stage breast cancer via miR140/Sox2/Sox9 pathway which is critical in stem cell renewal, differentiation, and cell migration in the tumor microenvironment [188].

It is true that the number of publications about the effects of brite/brown adipocytes on breast cancer is limited and the impacts of brite/brown adipocytes on breast cancer remain poorly understood. In the tumor microenvironment, brown/brite adipocytes impact positively on breast cancer. Enrichment of those cells in xenograft led to larger tumor size in mice [189]. Brown adipose tissue activity was reported to be significantly higher in breast cancer group, especially in young women with an increase of 25.6% compared to non-breast cancer group [190]. On the other hand, breast cancer cells can promote the differentiation of adipose stem cells in the tumor microenvironment [72].

## 5. Conclusions

**Figure 1:**
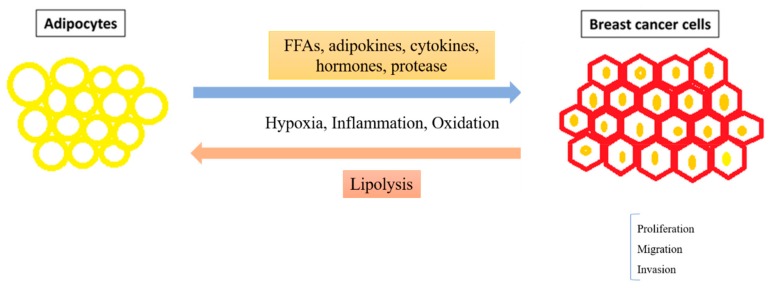
Adipocytes regulation of breast cancer.

Adipocytes are strongly linked to obesity-driven breast cancer through their secreted metabolic substrates, adipokines, and cytokines (Figure 1). Accumulated FFA, cholesterol, triglycerides, estrogen, leptin, insulin, interleukins, and chemokines together promote breast cancer initiation, proliferation, and invasion. In contrast, the adiponectin secreted by adipocytes is anti-tumorigenic in breast cancer. Evaluation of levels of each soluble factor secreted by adipocytes supports the prediction of the prognosis and anti-cancer treatment efficiencies in patients. Hyperinsulinemia induces insulin resistance in tumor and is linked to lower the IGF-1 expression and aromatase activity resulting in poor prognosis. In addition, obesity induces inflammatory microenvironment by adipocyte-released cytokines to promote cancer cell progression. Leptin, an adipokine, can de-stable HIF-1α and stimulate hypoxia condition in breast tumors. White adipose tissue can produce estrogen by aromatase, which is the most important enzyme for estrogen synthesis in obese post-menopausal women. High levels of estrogen in breast tissue promote cancer development and metastasis. Moreover, the estrogen-progesterone combination as a hormone replacement therapy or contraceptive preparation increases the incidence and mortality rate of breast cancer. Interestingly, tumor cells can regulate lipidation and lipolysis in adipocytes to provide energy and nutrients for tumor development [193]. Therefore, control of body weight, as well as weight gain in menopausal women, is necessary to reduce the risk of breast cancer.

## Figures and Tables

**Table 1:** Adipocytes regulate breast cancer via their metabolic substrates.

Metabolic Substrates		Released by White/Brite/Brown Adipocytes	Effect on BC Development	Effect on BC Cell Proliferation	Effect on BC Cell Invasion	References
Free fatty acids	Saturated; (n-6) fatty acids	White	Increase	Increase	Increase	[46,47,53,57,58,59]
	(n-3) fatty acids	White	Decrease	Decrease	Decrease	[43,45,49]
Lipids, Triglycerides		White	Increase	Increase	Increase	[61]
Cholesterol	Total	White	Increase	Increase	Increase	[60,62,63]
	HDL	White	Decrease	Decrease	Decrease	[44]
	LDL	White	Increase	Increase		[44]
	VLDL	White	Increase	Increase	Increase	[44]
	27-OHC	White	Decrease	Decrease		[41,53]
Exosome	mir- 3184-5p	White	-	Increase	Increase	[50]
	mir- 184c-3p	White	-	Decrease	Decrease	[50]
Proteases (MMP-9, MMP-11)		White	Increase	Increase	Increase	[74,75,76]

Note: 27-Hydroxycholesterol, 27-OHC; matrix metalloproteinase-9, MMP-9; high-density lipoprotein, HDL; low-density lipoprotein, LDL; very-low-density lipoprotein, VLDL; breast cancer, BC.

**Table 2:** Adipocytes regulate breast cancer via their released hormones.

Hormone	Released by White/Brite/Brown Adipocytes	Effect on BC Development	Effect on BC Cell Proliferation	Effect on BC Cell Invasion	Reference
Estrogen	White	Increase	Increase	Increase	[97,98,99,100,101]
Adiponectin	White	Decrease	Decrease	Decrease	[108,109,110,111,112,128]
Leptin	White	Increase	Increase	Increase	[111,112,113,114,118,119,128,132]
Insulin	White	Increase	Increase	Increase	[40,120,121,125,126]
Visfatin	White	Increase	Increase	Increase	[128]
PAI-1	White	Increase	Increase	Increase	[130,133]
Resistin	White	Increase	Increase	Increase	[128,129,134]
	White	Decrease	Decrease	Decrease	[135]

Note: Plasminogen activator inhibitor-1, PAI-1; breast cancer, BC.

**Table 3:** Adipocytes regulate breast cancer via released cytokines.

Hormone		Released by White/Bright/Brown Adipocytes	Effect on BC Development	Effect on BC Cell Proliferation	Effect on BC Cell Invasion	Reference
TNFα		Visceral, subcutaneous white	Increase	Increase	Increase	[132,143,144,145,147,148,149,150]
Interleukins	IL-1b	White	Increase	Increase	Increase	[142,157,158,159]
	IL-6	White	Increase	Increase	Increase	[132,138,141,142,143,161]
	IL-8	Visceral, subcutaneous white	Increase	Increase	Increase	[138,139,141,142,143]
	IL10	White				[138]
Chemokines	CCL2	White	Increase	Increase	Increase	[140,142]
	CCL5	White	Increase	Increase	Increase	[140,142]
	CXCL18	White				[169,170]
	CXCL12	White	Increase	Increase	Increase	[191,192]
	CXCL10/IP-10	White	Increase	Increase	Increase	[174,175]

Note: Interleukin, IL; C-X-C motif chemokine 10, CXCL10 or interferon γ-induced protein 10 kDa, IP-10; Tumor Necrosis Factor-alpha, TNFα; C-C Motif Chemokine Ligand 2, CCL2; breast cancer, BC.
